# Extensor Pollicis Brevis Tendon Transfer for Thumb Reconstruction in Radial Nerve Palsy: A Comparative Cadaveric Study

**DOI:** 10.1016/j.jhsg.2025.100781

**Published:** 2025-07-29

**Authors:** Yosuke Ito, Yusuke Matsuura, Takane Suzuki, Takahiro Yamazaki, Kenji Kubota, Seiji Ohtori

**Affiliations:** ∗ Department of Orthopaedic Surgery, Graduate School of Medicine, Chiba University, Chuo-ku, Chiba, Japan

**Keywords:** Extensor pollicis brevis, Radial nerve palsy, Tendon transfer, Thumb abduction, Tsuge method

## Abstract

**Purpose:**

In this study, we proposed a new tendon transfer method for thumb function reconstruction to treat radial nerve injuries. We specifically focused on enhancing thumb radial abduction by transferring the palmaris longus (PL) to the extensor pollicis brevis (EPB) while preserving the first compartment.

**Methods:**

Eight freshly frozen cadaver specimens were used to compare our proposed technique (transferring the PL to the EPB while preserving the first compartment) with the Tsuge method (transferring the extensor pollicis longus (EPL) and fixing the abductor pollicis longus to the flexor carpi radialis). Thumb radial deviation, palmar abduction, and interphalangeal joint extension angles were measured at various traction forces.

**Results:**

This method demonstrated superior efficiency in thumb radial abduction (especially at traction forces of 15 N and 20 N) compared to the Tsuge method.

**Conclusions:**

Using the proposed method, the thumb extension and abduction functions can be reconstructed without requiring a pulley. However, concerns were raised about potential inadequate interphalangeal joint extension, although this can be mitigated by suturing the EPB and EPL. Our findings indicate that this method is suitable for our biomechanics study, suggesting its potential applications for cases in which radial nerve injuries necessitate tendon transfer.

**Clinical relevance:**

The proposed method of transferring the PL to the EPB achieves more effective radial deviation of the thumb than the Tsuge method, highlighting its clinical applicability.

Tendon transfer is the treatment of choice for irreversible radial nerve palsy when nerve repair or transfer is not feasible. Approximately 50 tendon transfer procedures have been reported for treating radial nerve injuries.^1^ Despite these options, achieving optimal thumb function remains challenging, particularly with respect to thumb radial abduction.

In Riordan’s classical technique, the pronator teres is transferred to the extensor carpi radialis brevis, the flexor carpi ulnaris (FCR) to the extensor digitorum communis, and the palmaris longus (PL) to the extensor pollicis longus (EPL).^2^ Although this approach is effective for many aspects of hand function, it often results in suboptimal thumb radial abduction. Tsuge^3^ introduced modifications to address this issue by fixing the abductor pollicis longus (APL) to the FCR and using it as a pulley. However, clinical observations have shown insufficient thumb radial abduction force in cases treated using the Tsuge method.^4^ Kruft et al^5^ reported an alternative method by preserving the first compartment, which achieved good thumb abduction but made isolated thumb extension challenging.

Both effective thumb radial abduction and controlled extension are challenging to achieve using the existing techniques. Herein, we proposed a novel method that preserves the first compartment to obtain radial abduction of the thumb while transferring the PL to the extensor pollicis brevis (EPB), allowing for isolated thumb extension. We expect this new method to result in a more efficient radial deviation of the thumb than the Tsuge method. Furthermore, we compared and evaluated the effectiveness of these thumb extension and abduction reconstruction techniques using freshly frozen cadavers.

## Materials and Methods

This study was conducted between June 2022 and June 2023. A total of eight upper limbs were collected from six cadavers, including three males and three females, with an average age of 90 years (range: 85–97 years). Upon visual inspection, any specimen showing signs of previous trauma, scars, and deformities was excluded. Each arm was severed at the proximal half of the humerus and thawed at room temperature (20 °C) just before the experiment. During the procedure, hydration was maintained by spraying saline solution. The wrist was fixed in the neutral position (0° flexion/extension, 0° radial/ulnar deviation), and the forearm was fixed in neutral rotation. Ethical approval for this study was obtained from Ethics Committee of Chiba University Graduate School. Written informed consent was obtained from all subjects before the study.

**Figure 1:**
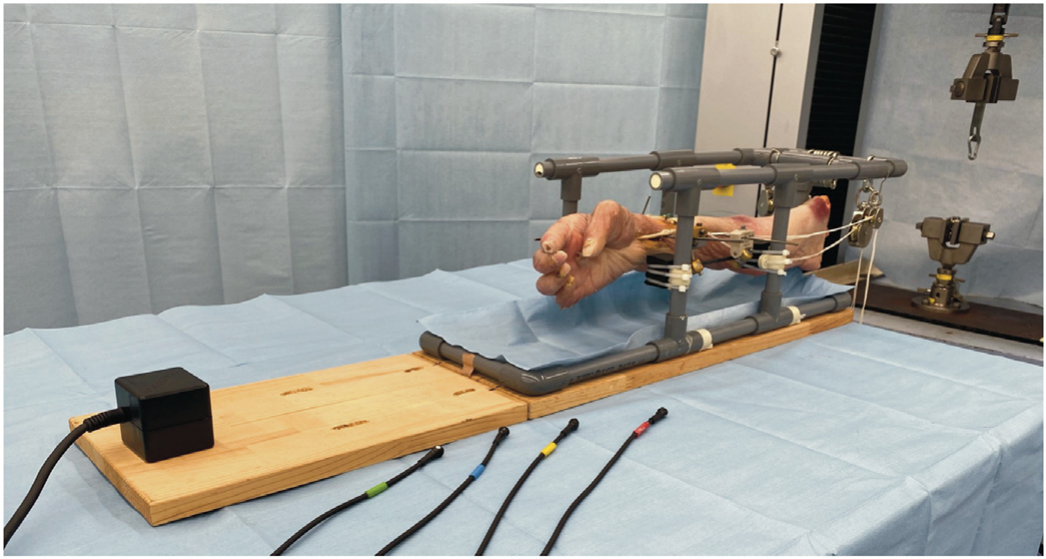
Apparatus set-up. The specimen is secured to the testing apparatus, including a 3-dimensional electromagnetic motion tracking system, a fixture, and a mechanical testing machine.

**Figure 2:**
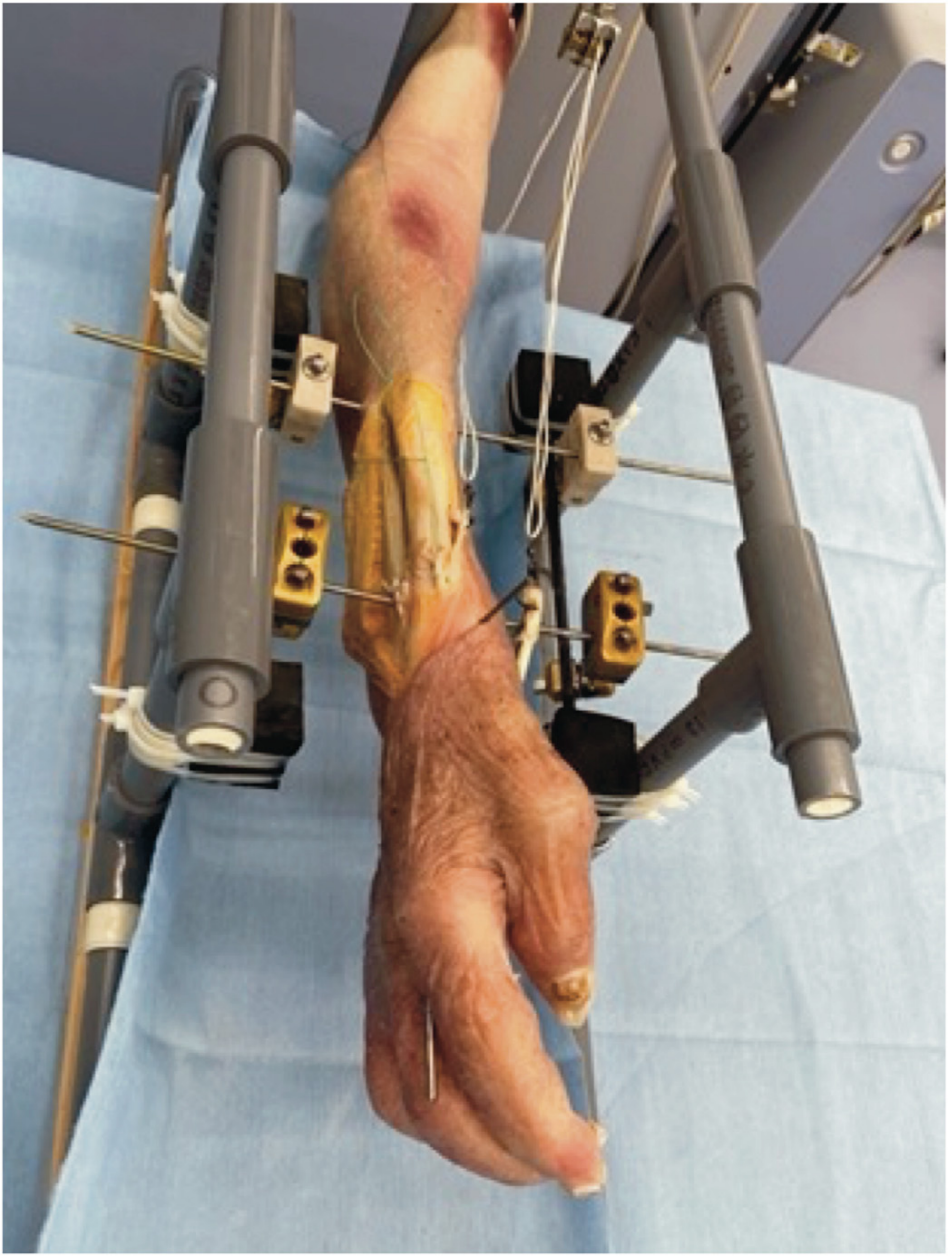
Arm positions. The arm is secured with Kirschner wires, and the wrist and forearm are fixed in the neutral position.

To secure the specimens during testing, we attached the upper limbs to a custom fixture constructed using nonmagnetic materials, including resin-filled polyethylene vinyl pipes, carbon rods, wood, and an magnetic resonance imaging-compatible external fixator. The radiocarpal joint, distal radioulnar joint, and radial shaft at the wrist were fixed in the positions of wrist palmar/dorsal flexion, radial/ulnar deviation, and forearm pronation/supination, respectively, using titanium Kirschner wires (Figure 1, Figure 2). The initial tension was established by pulling the FPL proximally with a 200-g load.

Because this study was conducted on cadavers, we could not directly measure the actual physiological pulling force of the tendons. However, based on previous reports, we calculated the estimated muscle forces and conducted the study with a maximum traction force of 25 N to minimize the impact on the soft tissues. The previously estimated muscle forces for PL, EPL, and EPB were approximately 15.5–40.5 N, 22.05–42.75 N, and 10.575 N, respectively.^6^, ^7^, ^8^ The PL is used as a donor in the new and Tsuge methods. Because the muscle force of the donor muscle is larger than that of the recipient, the principles of tendon transfer are satisfied.

### Clinical application of the surgical technique

#### The new method

**Figure 3:**
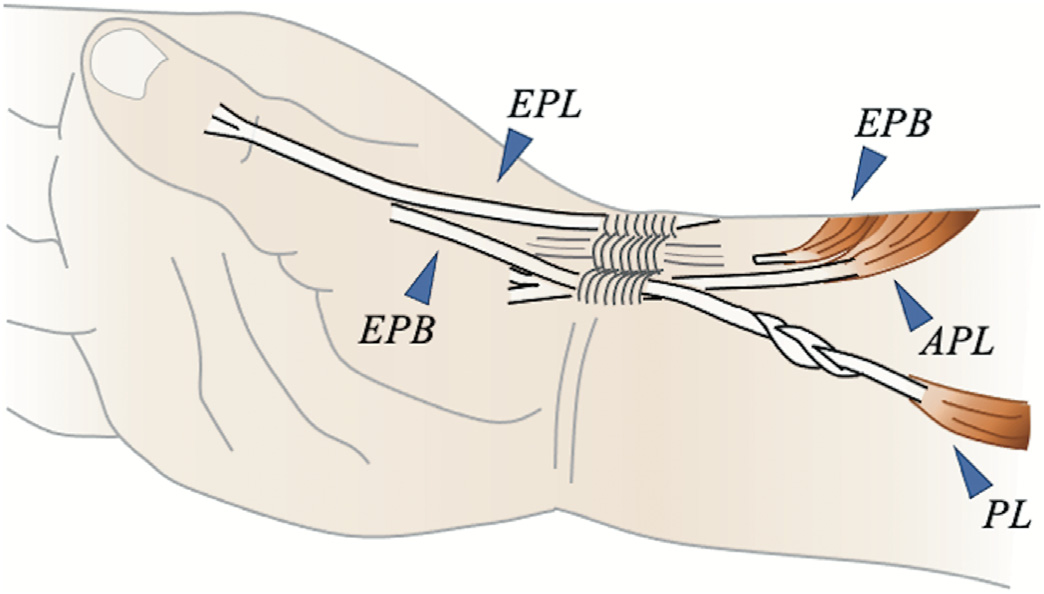
Schematic diagram of the proposed surgical technique in a clinical setting. The EPB was detached at the myotendinous junction, and a 3- to 4-weave interlacing suture was performed between the EPB and PL.

To implement the new method, we first confirmed the presence of the EPB in the first compartment slightly proximally and detached it at the myotendinous junction on the dorsal side of the distal forearm (Fig. 3). If the thumb interphalangeal (IP) joint extension angle did not reach 0° after pulling the EPB, we sutured the EPB and EPL on both sides at the level of the metacarpophalangeal (MP) joint. An incision was then made on the palmar side of the wrist to secure the PL and guide it proximally to the first compartment. A 3-to 4-weave interlacing suture was applied between the EPB and PL while applying sufficient tension.

#### The Tsuge method

**Figure 4:**
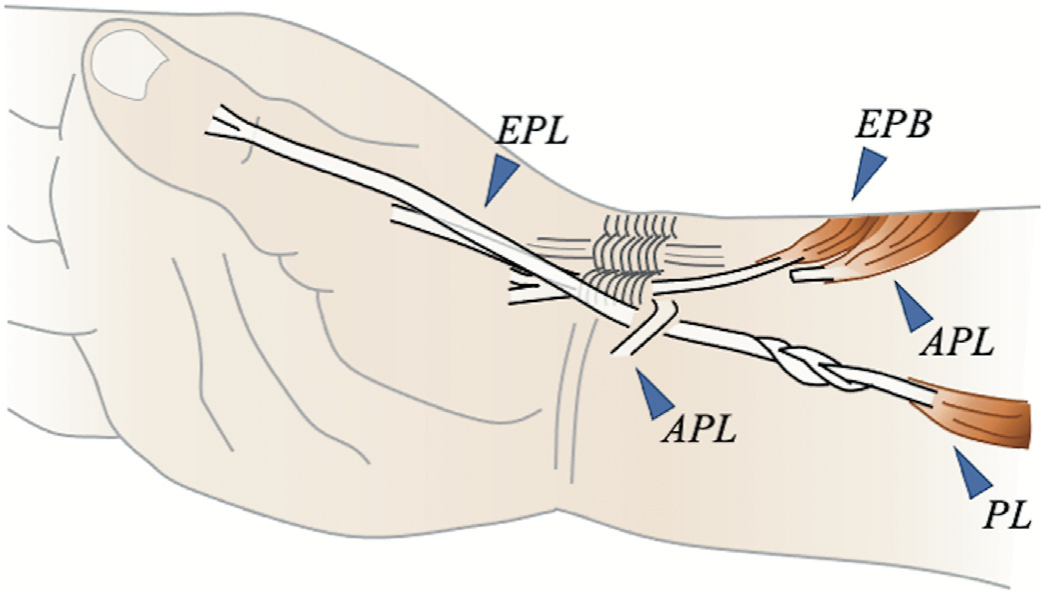
Schematic diagram of the surgical technique using the Tsuge method in a clinical setting. The third compartment was opened to detach the EPL at the myotendinous junction. Then, the APL was cut proximally at the first compartment, pulled, and fixed to the FCR in thumb abduction, creating a pulley. The EPL was moved to the palmar side, passing through the previously created pulley and performing an interlacing suture with the PL.

To execute the Tsuge method, we opened the third compartment, detached the EPL at the myotendinous junction, and made an incision on the palmar side to secure the PL (Fig. 4). The APL was cut proximally at the first compartment, pulled, and fixed to the FCR in thumb abduction to create a pulley in this portion. The EPL was moved to the palmar side and passed through the previously created pulley, and an interlacing suture was performed with the PL.

### Research model

#### The EPB group

**Figure 5:**
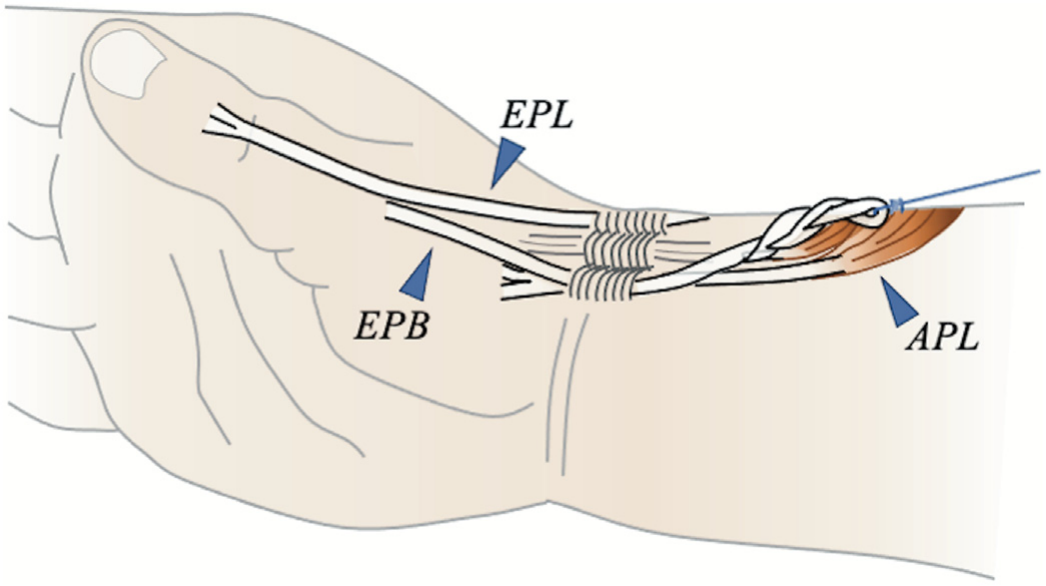
Schematic diagram of the EPB group. The EPB was detached at the myotendinous junction, creating a loop at its proximal end, which was firmly sutured. A fluorocarbon thread was passed through the loop, and the EPB was pulled dorsally toward its attachment using a mechanical testing machine.

For the EPB group, we made an incision on the dorsal side of the wrist and detached the EPB at the myotendinous junction (Fig. 5). A loop was created at the proximal end of the EPB and firmly sutured with a 4-0 nonabsorbable braided suture. By passing a fluorocarbon thread through the loop, the EPB was pulled dorsally toward its attachment using a mechanical testing machine to reproduce the physiological muscle contraction of the EPB.

#### The EPL group

**Figure 6:**
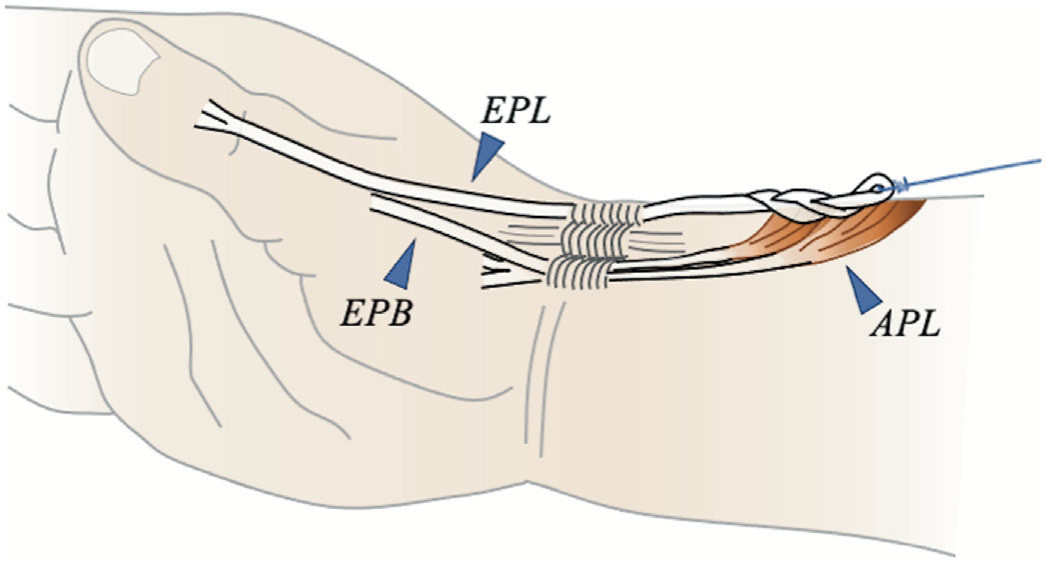
Schematic diagram of the EPL group. The EPL was detached at the myotendinous junction. Using the mechanical testing machine, the same loop and thread treatment was applied as in the extensor pollicis brevis group to pull the EPL dorsally toward its attachment.

For the EPL group, an incision was made on the dorsal side of the wrist to detach the EPL at the myotendinous junction (Fig. 6). Using the same loop and thread method as described above, the EPL was pulled dorsally toward its attachment using the mechanical testing machine to reproduce its physiological muscle contraction.

#### The New method

**Figure 7:**
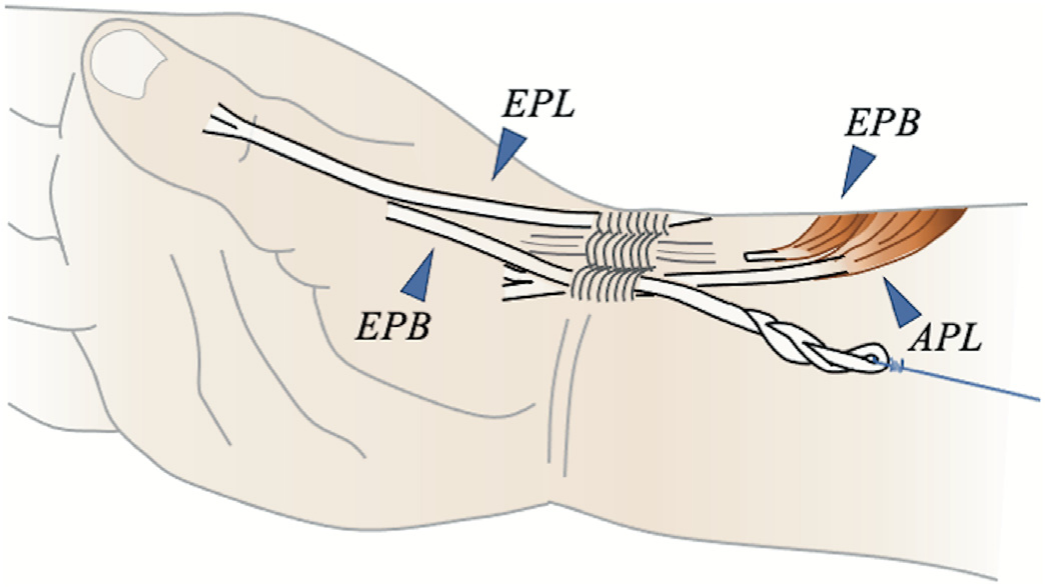
Schematic diagram of the proposed method. The loop model created in the EPB group was moved dorsally. Using a fluorocarbon thread, the EPB was pulled toward the attachment of the PL at the inner side of the proximal humerus using the mechanical testing machine.

To execute the proposed method, we made an incision on the palmar side of the wrist and moved the loop model created in the EPB group dorsally (Fig. 7). Using a fluorocarbon thread, we pulled it toward the attachment of the PL at the inner side of the proximal humerus using the mechanical testing machine. In cases where the EPB was pulled and the thumb IP joint extension angle did not reach 0° (which occurred in two out of eight cases), we sutured the EPB and EPL on both sides at the MP joint level.

#### The Tsuge method

**Figure 8:**
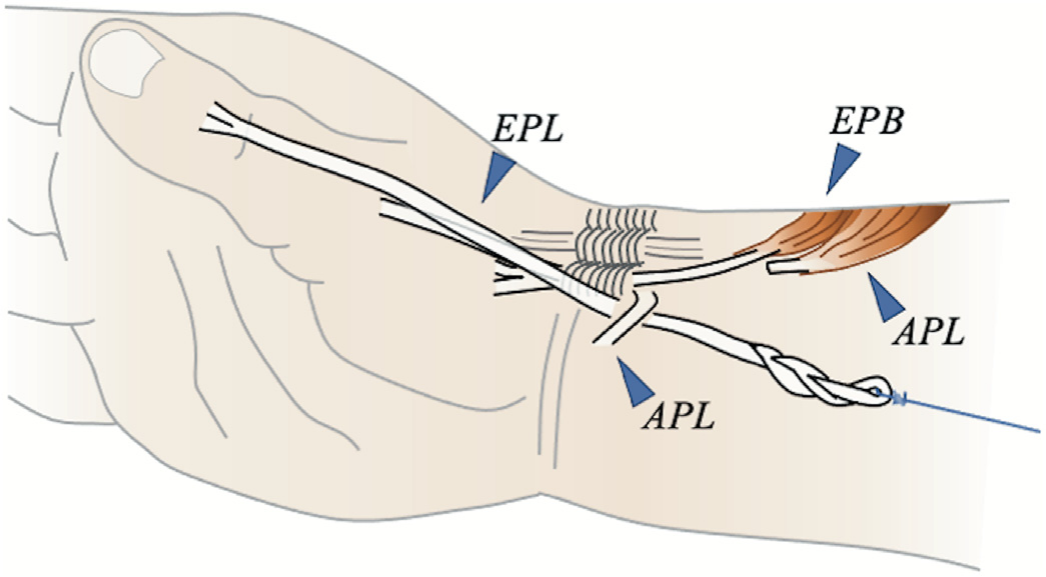
Schematic diagram of the Tsuge method. The third compartment created in the EPL group was opened to detach the EPL from the third compartment. The APL was cut proximally at the first compartment. Then, it was pulled while fixing it to the FCR in thumb abduction, creating a pulley. The EPL was passed through this pulley and then pulled dorsally on the inner side of the proximal humerus using the mechanical testing machine.

To reproduce the Tsuge method, we opened the third compartment created in the EPL group to detach the EPL, which was pulled out from the palmar incision site (Fig. 8). Next, we cut the APL proximally at the first compartment, pulled it while fixing it to the FCR in thumb abduction, and created a pulley in this portion. The EPL was passed through this pulley and pulled dorsally on the inner side of the proximal humerus using the mechanical testing machine.

Table 1 provides a comparative overview of these techniques, illustrating the donor–recipient relationship of the tendon transfers and the differences in the preserved anatomical structures across each method. In this study, the EPB and EPL were pulled in the directions of their respective muscle attachments to reproduce physiological contractions. Data measurements were then taken after reproducing each technique on eight upper limbs, performing four techniques on a single upper limb.

**Table 1:** Comparison of the Tendon Transfer Methods Evaluated in this Study

Method	Donor Tendon	Recipient Tendon	Preserved Structures	Key Features
Proposed method	Palmaris longus (PL)	Extensor pollicis brevis (EPB)	First compartment	Preserves the natural pulley system; Enables isolated thumb extension
Tsuge method	Palmaris longus (PL)	Extensor pollicis longus (EPL)	None	Creates an artificial pulley using APL fixed to FCR
EPB group (control)	N/A (direct traction)	Extensor pollicis brevis (EPB)	First compartment	Evaluates natural EPB biomechanics
EPL group (control)	N/A (direct traction)	Extensor pollicis longus (EPL)	Third compartment	Evaluates natural EPL biomechanics

N/A, not applicable.

### Measurements

**Figure 9:**
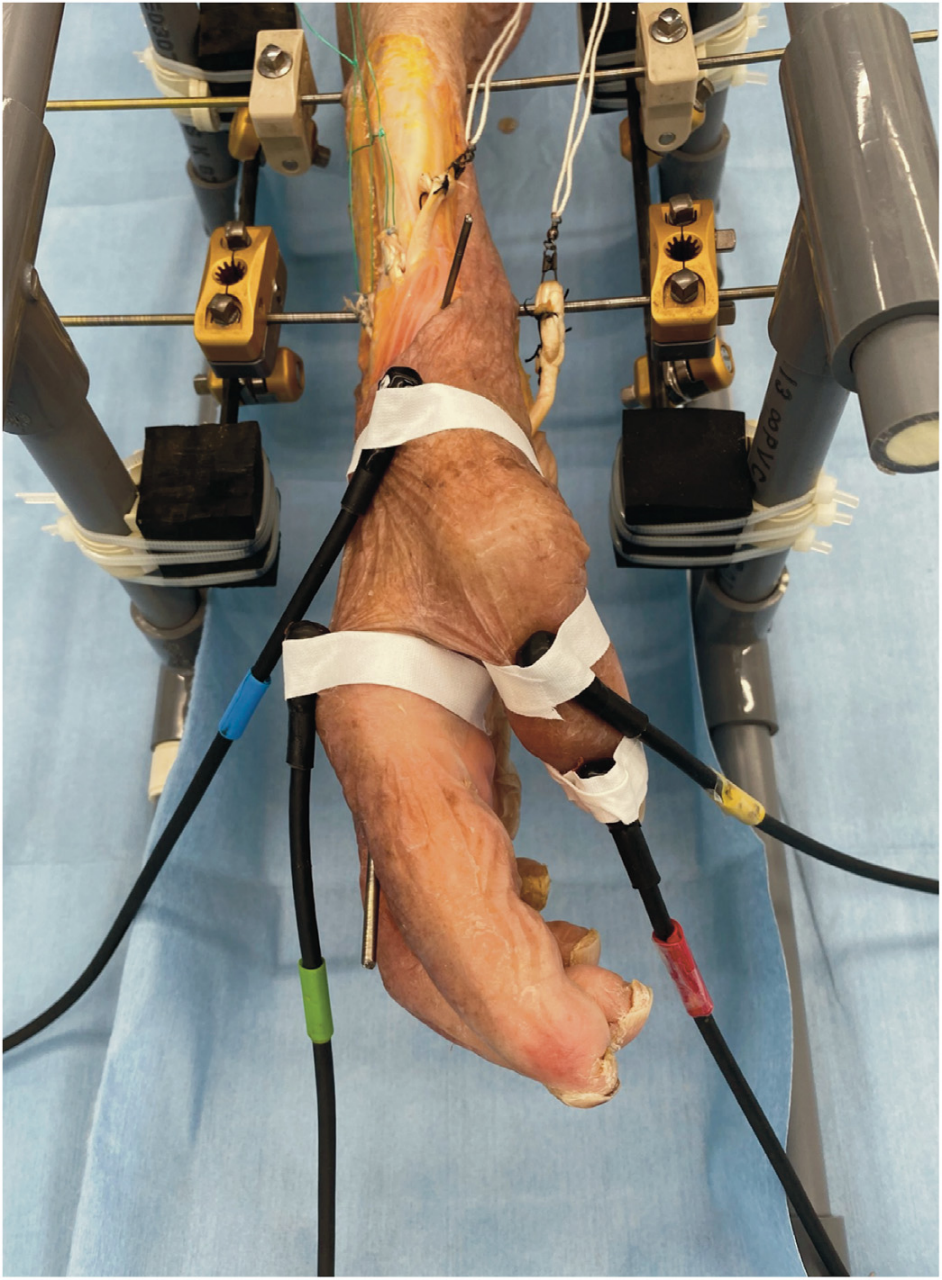
Positions of the sensors. Sensors were attached to the thumbnail, the bases of the first metacarpal bone and the first proximal phalanx, and the dorsum of the head of the second metacarpal bone.

A 3-dimensional electromagnetic motion tracking system was used for spatial analyses. In this system, the sensor position and orientation are consistently measured and recorded in a 40 Hz electromagnetic field. Sensors were attached to the thumbnail, the bases of the first metacarpal bone and the first proximal phalanx, and the dorsum of the head of the second metacarpal bone, and their positions in 3D space were recorded (Fig. 9). The tension generated during tendon traction was recorded in terms of the force (N) and the 3-dimensional coordinates of the sensors using a mechanical testing machine.

The angles of the radial deviation, palmar abduction, and IP joint extension of the thumb were measured based on the 3-dimensional coordinates. The angles at forces of 0, 5, 10, 15, 20, and 25 N were recorded. Traction forces of 0, 5, 10, 15, 20, and 25 N were applied to test the biomechanical properties. These values were selected based on previous studies, which estimated the physiological muscle forces for PL (15.5–40.5N), EPL (22.05–42.75 N), and EPB (10.575 N).^6^, ^7^, ^8^ The maximum traction force was limited to 25 N to minimize tissue damage while ensuring clinically relevant force ranges.

Regarding the thumb IP joint extension angle, a subgroup excluding specimens whose IP joint extension angles did not reach 0°, even after EPB traction (2/8 cases), was analyzed separately as the EPB ex group.

### Statistical analysis

The differences in the angles of the radial deviation, palmar abduction, and IP joint extension of the thumb between the proposed and Tsuge methods were compared using paired-sample *t* tests. Statistical significance was set at *P* < .05.

## Results

### Thumb radial deviation angle

**Figure 10:**
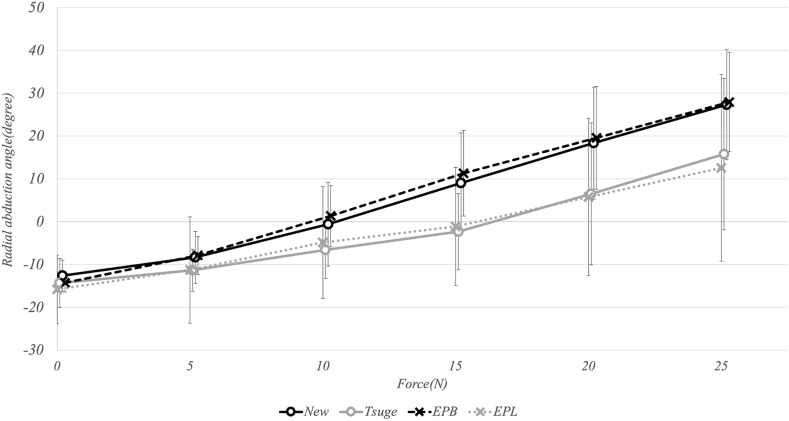
Graphical representation of the thumb radial abduction angles in relation to the traction force. The thumb radial abduction angle in the new method was larger at all traction forces. Additionally, there was no significant difference in the thumb radial abduction angle between the new method versus the EPB group and the Tsuge method versus the EPL group.

In the specimens operated on using the new method, the thumb radial abduction angle was turned to the radial side at a traction force of 10 N, whereas a traction force of 20 N was required for radial abduction in the Tsuge method. Although the thumb radial abduction angle in the new method was larger at all traction forces, this difference was significant only at 15 N (*P* = .04) and 20 N (*P* = .04) (Fig. 10). At 25 N, the angles reached 27.3° and 15.7° for the new and Tsuge methods, respectively. Additionally, no significant difference was seen in the thumb radial abduction angle between the new method versus the EPB group and between the Tsuge method versus the EPL group, despite the said angles being larger in the new method and the EPB group than in the Tsuge method and the EPL group.

### Thumb IP joint extension angle

**Figure 11:**
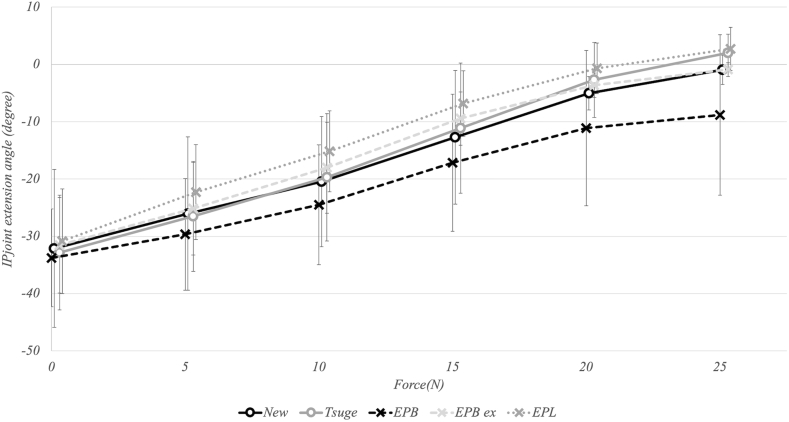
Graphical representation of the thumb IP joint extension angle in relation to the traction force. As the traction force increased, the thumb IP joint extension angle increased in the new and the Tsuge methods without any statistically significant differences between both groups.

At 25 N, the thumb IP joint extension angle was −0.9° for the new method and 2.0° for the Tsuge method, achieving near-extension at 0°. As the traction force increased from 0 N to 25 N, the thumb IP joint extension angle increased steadily for both methods (new method: from −32.1° to −0.9°; Tsuge method: from −32.8° to 2.0°) (Fig. 11). There was no statistically significant difference in the thumb IP joint extension angle between the new and Tsuge methods at any traction force level (*P* > .05 for all comparisons). The EPL group tended to have a larger thumb IP joint extension angle than the other three groups, reaching 2.7° at 25 N compared to −0.9° (new method), 2.0° (Tsuge method), and −0.9° (EPB ex group).

### Thumb palmar abduction angle

**Figure 12:**
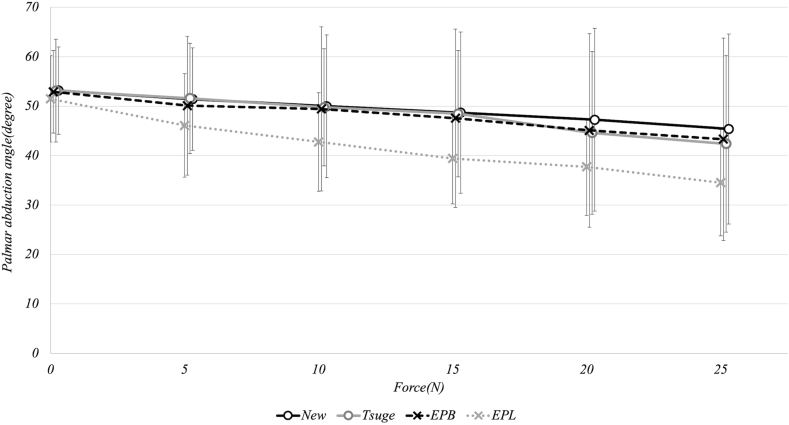
Graphical representation of the thumb palmar abduction angle in relation to the traction force. The palmar abduction angle was decreased as the traction force increased in the groups operated on using the new and Tsuge methods, without any significant difference between the groups.

The specimens operated on using both methods showed a decrease in the palmar abduction angle as the traction force increased, without any significant difference between groups. At 0 N, the palmar abduction angles were the same (53.1°) for both methods (Fig. 12). These angles decreased to 45.3° (new method) and 42.3° (Tsuge method) at 25 N, with no statistically significant differences at any traction force level (*P* > .05). Moreover, in all four groups, as the traction force increased, the palmar abduction angle decreased in a similar pattern: from 52.9° to 43.3° in the EPB group and from 51.4° to 34.5° in the EPL group (0 N to 25 N). The subjects in the EPL group tended to have a smaller palmar abduction angle at higher traction forces than the other groups.

## Discussion

We developed a novel method for transferring tendons from the PL to the EPB. The main advantages of the proposed procedure are summarized as follows:

(1) EPB, compared to EPL, performs more radial abduction of the thumb: A comparison of the radial abduction angles of the models with EPB traction (the new method vs EPB group) and those with EPL traction (Tsuge method vs the EPL group), the model with EPB traction showed a larger tendency. Our biomechanical testing demonstrated that the new method provides superior efficiency in thumb radial abduction compared to the Tsuge method. Specifically, at clinically relevant traction forces of 15 N and 20 N, our method achieved significantly greater radial deviation angles. This is likely because EPB inherently performs purely radial deviation of the thumb, making it advantageous for thumb radial abduction. Therefore, in Riordan’s and Tsuge’s methods (where the source of radial abduction of the thumb is the EPL), the new method is more advantageous for radial abduction.
(2) Efficient reconstruction of thumb extension and abduction function: Regarding the efficient reconstruction of thumb extension and abduction function, the new method uses a pulley for tendon transfer in the physiological native first compartment. Furthermore, beyond the pulley, it follows the natural course of the EPB (eliminating the need for an extension at the same site), and there is no concern about adhesion. Therefore, no increase in the sliding resistance in the pulley or beyond is expected, enabling the efficient transmission of force. In contrast, with the Tsuge method, the APL is detached and fixed to the FCR in the extended position, which may increase the sliding resistance to the nonphysiological pulley.
(3) A pulley is not required: The absence of the need to create a pulley is related to the Tsuge method, where the APL is detached and fixed to the FCR in the extended position, which may cause the pulley itself to loosen compared to the present method using the natural pulley for tendon transfer. In the present method, using the natural pulley for tendon transfer, the pulley effect is expected to be fully exerted.
(4) Prevention of thumb palmar abduction because of thumb extension, allowing pure thumb extension and radial abduction: As thumb extension is the inherent action of the EPB when pulled, the EPB does not induce palmar abduction. In Riordan’s and Tsuge’s methods, the EPL is released from the third compartment and moves to the palmar side. Resultantly, the EPL operates in a more palmar manner than the EPB, making it mechanically difficult to achieve radial deviation. This leads to postoperative complaints of insufficient thumb radial deviation. However, in the new method, palmar abduction can be performed by the intrinsic muscles of the thumb under the control of the median nerve (the short abductor muscle of the thumb, the short flexor muscle of the thumb, and the opponens pollicis muscle). Therefore, theoretically, functions other than retropulsion (which are the pure functions of the EPL) can be exerted.

Inadequate IP joint extension is a potential drawback of the new method. Takahashi et al^9^ reported that the EPB attaches to the base of the proximal phalanx, distal phalanx, and extensor tendon cap in 20%, 34%, and 46% of the cases, respectively. Sugiura et al^10^^,^^11^ reported that 43% to 50% of EPBs are attached to the distal phalanx and that IP joint extension is possible. Moreover, cases have been reported where IP joint extension could be achieved despite EPL rupture by pulling the EPB, even in cases where it attaches to the extensor tendon cap.^12^^,^^13^ In this study, in two out of eight cases (25%), the IP joint angle was less than 0° when EPB was pulled. However, in both cases with IP joint extension insufficiency, an IP joint extension of at least 0° was achieved by suturing the EPB and EPL on both sides. Even when using EPB for transfer, IP joint extension insufficiency can be avoided. The research findings showed no significant difference in the thumb IP joint extension angle between the new and Tsuge methods, and both achieved good extension. Moreover, in the Merle d'Aubigne method, achieving sole extension of the thumb is challenging.^5^ However, it is possible in all cases to use the new method by performing additional procedures.

This study has several limitations. First, we did not analyze the sensitivity of the 3D electromagnetic motion tracking system or perform a prior power analysis to determine the appropriate sample size. We also could not randomize the measurement sequence. These methodological limitations may affect the reliability and validity of our results. Second, the study was conducted only on cadavers. Although we simulated tendon function through mechanical traction, this may not fully recapitulate the physiological muscle contraction patterns seen in vivo. The constant directional pull applied by the mechanical testing machine differs from the dynamic nature of the natural muscle contractions. Third, the test performed in this study is a single-pull traction test, and it does not evaluate slack or long-term deviation of tendon movement that may occur clinically. Especially in the Tsuge method, in which a pulley is created, the outcome may have been favorable. Fourth, adhesions were not evaluated because this study was not conducted on live patients. Although the proposed procedure is expected to have minimal impact on adhesion, this could not be verified.

Patterson et al^14^ compared the clinical outcomes of tendon and nerve transfers, showing that both approaches effectively improved grip strength, pinch strength, function scores, and quality of life scores. However, their study did not specifically evaluate the thumb radial abduction function in detail. Future clinical studies using the proposed technique should include a detailed assessment of thumb radial abduction and compare outcomes with established procedures to validate the biomechanical advantages observed in this cadaveric study.

## Conflicts of Interest

No benefits in any form have been received or will be received related directly to this article.
